# Free Thyroxine Level in the High Normal Reference Range Prescribed for Nonpregnant Women May Reduce the Preterm Delivery Rate in Multiparous

**DOI:** 10.4061/2011/905734

**Published:** 2011-12-12

**Authors:** P. Torremante, F. Flock, W. Kirschner

**Affiliations:** ^1^Praxis für Gynäkologie und Geburtshilfe, Marktplatz 29, 88416 Ochsenhausen, Germany; ^2^Frauenklinik des Klinikums Memmingen, Klinikum Memmingen, Bismarckstraße 23, 87700 Memmingen, Germany; ^3^Institut Forschung, Beratung und Evaluation, FB+E Forschung, Beratung Evaluation GmbH, Postfach 10 03 35, 10563 Berlin, Germany

## Abstract

Preterm birth is the most common reason for perinatal morbidity and mortality in the western world. It has been shown that in euthyreotic pregnant women with thyroid autoimmune antibodies, L-Thyroxine replacement reduces preterm delivery rate in singleton pregnancies. We investigated in a nonrandomized retrospective observational study whether L-Thyroxine replacement, maintaining maternal free thyroxine serum level in the high normal reference range prescribed for nonpregnant women also influences the rate of preterm delivery in women without thyroid autoimmune antibodies. As control group for preterm delivery rate, data from perinatal statistics of the State of Baden-Württemberg from 2006 were used. The preterm delivery rate in the study group was significantly reduced. The subgroup analysis shows no difference in primiparous but a decline in multiparous by approximately 61% with L-Thyroxine replacement. Maintaining free thyroxine serum level in the high normal reference range prescribed for nonpregnant women may reduce the preterm delivery rate.

## 1. Introduction

The prevalence of preterm birth, defined as delivery before 37 completed weeks' gestation, varies from 6% to 15%, depending on geographical and demographic features of the population studied. Preterm births account for 75% of perinatal deaths, with over two-thirds of these arising in preterm infants delivered before 32 weeks' gestation. In 2002, this was the most frequent cause of neonatal deaths in the USA. 65%–75% of preterm births are defined as spontaneous preterm births caused by spontaneous preterm labor or preterm premature rupture of the membranes and 30%–35% of preterm deliveries are medically indicated due to maternal or fetal complications in pregnancy. About one-quarter of preterm births occur in multiple pregnancies [1–5]. 

Various epidemiological risk factors were identified. These encompass advanced age or teenage pregnancy, parity, race, history of cervical cone biopsy, low body mass index, tobacco use, assisted conception with in vitro fertilisation, and gamete intrafallopian transfer, especially for singleton gestations, systemic and genital-tract infection, and low parental socioeconomic status, this being the most important factor [1–4, 6]. Thanks to advances in neonatal medicine, the outcome for preterm infants born at or after 32 weeks' gestation is similar to that of full-term infants. Limits of viability have been lowered to a gestational age (GA) as low as 23 weeks at the expense of physical disabilities and long-term neurodevelopmental consequences [2, 4, 5, 7].

Thyroid hormones are essential for differentiation and maturation of the fetus and placenta and are especially important for the development of the fetal central nervous system. Epidemiological studies have demonstrated that the intelligence quotient (IQ) of the offspring correlates with maternal free thyroxine (fT4) serum level. The higher maternal fT4, the higher the IQ of the offspring [8–13]. In a study with euthyroid thyroid peroxidase antibody (TPOAb) positive pregnant women receiving substitution with L-Thyroxine (L-T4) it proved possible to decrease their preterm birth rate [14–16]. Thyroid hormones may also provide protection against preterm delivery in women without TPOAb.

The aim of this study is to investigate whether L-T4 replacement therapy by maintaining maternal fT4 in the high normal reference range prescribed for nonpregnant women can also lower the preterm birth rate in pregnant women who do not have thyroid autoimmune antibodies. For this purpose, a group of pregnant women, who had been treated with L-T4 with the goal of elevating maternal fT4 serum level to the high normal reference range prescribed for nonpregnant women to achieve an optimal fetal supply with thyroxine, were retrospectively examined.

## 2. Subjects and Methods

This clinical trial is a retrospective nonrandomized observational study with prospectively designed data. From April 2001 to March 2010, all pregnant women below 12 weeks' gestation presenting for their first medical consultation for prenatal care in a medical office were offered a serological thyroid scan. This included basal TSH, free triiodothyronine (fT3), free thyroxine (fT4), thyroglobulin antibody test (TgAb), thyroid peroxidase antibody test (TPOAb), and TSH receptor antibody test (TSH-R-Ab).

The primary object was to elevate maternal fT4 to the high normal reference range for nonpregnant adults in order to optimise fetal brain development. For this purpose, and for better interpretation, the normal reference range for nonpregnant adults (fT4 12.14–19.62 pmol/L) was divided into thirds. The division was made as follows: the lower third ranged from 12.14–14.72 pmol/L, the middle third ranged from 14.72–17.17 pmol/L, and the upper third ranged from 17.17–19.62 pmol/L. All women with an fT4 serum level in the lower and middle third of the reference range for nonpregnant adults at the first consultation were given a variable dose of L-T4, usually starting with 25 *μ*g–50 *μ*g L-T4 per day, after informed consent, to raise the fT4 serum level in the high normal reference range (upper third) for nonpregnant adults. All women with a physiological fT4 serum level in the high normal reference range (upper third) for nonpregnant adults at the first consultation were only serological assessed and L-T4 therapy was started later when serum fT4 declined to the middle or lower third. Additionally, each woman received supplements containing 200 *μ*g iodide and 400 *μ*g folic acid.

Maternal fT4 serum levels were regularly assessed after an interval of 24 hours from the last L-T4 intake every 4 weeks during routinely performed serological scans for pregnancy care and if necessary, the L-T4 dose was adjusted. For adjustment, a variable dosage for example, between 25–50 *μ*g/die L-T4 was usually applied. If the increased dose was not tolerated, it was recommended to continue with the last tolerated dose and to increase it one week later. TSH was not further assessed because the goal was to avoid a low maternal fT4 serum level in pregnancy for optimal fetal brain development, and a decline of the fT4 serum level is not automatically accompanied by an increase of the TSH level. Furthermore, maternal TSH is not related to the cognitive competence of the offspring [12]. Moreover, in pregnancy, TSH shows dependencies with other pregnancy-associated hormonal fluctuations and the interval between two blood samples of 4 weeks is too short for precise TSH readings [17]. Thyroid function control was handled by determining fT4 and not TSH as is the case when assessing central hypothyroidism [18–20].

Prenatal care included measurement of body weight, blood pressure, urinary test for protein, glucose, blood, and nitrite. Check intervals were as follows: every 4 weeks until the 30 weeks' gestation, thereafter every 3 weeks, weekly after the 35 weeks' gestation, and after term every 2 days. Beginning at 20 weeks' gestation a digital vaginal examination was performed, and if necessary an ultrasound scan of the cervix length was done.

Ultrasound scans were performed between gestation weeks 9–12, 19–22, and 29–32. If required, color Doppler sonography and special ultrasound scan to rule out fetal malformation were undergone. A cardiotocographic survey of the fetus was routinely started at 28 weeks' gestation and thereafter at each visit.

Women who developed preterm labor were treated with orally applied magnesium. In case of hospitalisation, treatment of preterm labor was performed according to hospital guidelines, for example, bed rest, intravenous fluids, tocolytic therapy, and steroid administration, if clinically indicated. Administration of L-T4 was continued during treatment for preterm labor.

All pregnant women were delivered in an obstetric unit in different hospitals in the vicinity. Routinely, the newborns were medically examined by the gynaecologist who had assisted parturition immediately after birth. Apgar score and arterial blood gas analysis from the umbilical cord were measured. A further examination of the newborn was performed by a paediatrician between the 3rd–7th day of life. After discharge, all children in Germany have regular medical checkups performed by a paediatrician or family physician in private office in regular intervals beginning at 4–6 weeks after birth until adolescence. 6–8 weeks postpartum all women had a medical check for controlling uterine involution and after establishing normal serum fT4 levels, L-T4 substitution was discontinued.

The study group included all women who fulfilled the following criteria: women with singleton pregnancies who had their first antenatal check before the 12 weeks' gestation, determined by ultrasound (crown-rump length). The women presented regularly for inspection until birth. Since this clinical trial was designed as a retrospective nonrandomized observational study, we chose a surrogate as control group. The study was performed in the State of Baden-Württemberg, so we used as control group the preterm delivery rate from all 87.897 singleton pregnancies of the State of Baden-Württemberg, Germany, in the year 2006, collected in central database (GeQiK) with unknown thyroid status. The processed data, provided to us by the database GeQik of the perinatal statistics of the State of Baden-Württemberg were related to preterm birth rate, maternal age and parity status. A further comparison of more obstetrical details and perinatal outcomes was not possible to realize. Preterm delivery was defined as parturition before completion of the 37 weeks' gestation.

## 3. Laboratory Analysis

Serum basal TSH and fT4 were measured using a third generation electrochemiluminescence immunoassay (Elecsys 1010/2010–MODULAR ANALYTICS E170 from Roche Diagnostics GmbH–Mannheim, Germany). Reference values for TSH were 0.27–4.2 *μ*IU/mL and for fT4 12.14–19.62 pmol/L. Intra- and interassay coefficients of variation were 3.0% and 7.2% for TSH, and 1.4% and 3.5% for fT4. Thyroid antibodies were also determined using the above-mentioned analytic test. Thyroid antibody titers were considered positive for TPO-Ab titers above 34 U/mL, for Tg-Ab titers above 115 U/mL, and for the anti-TSH receptor-Ab titers above 2.0 U/L.

## 4. Statistical Analysis

Basis for data of the control group are population parameters statistical analysis took place by calculating the 99% confidence intervals (99%-CI) of the values in the study group.

## 5. Results

Between April 2001 and March 2010, 771 pregnant women presented for medical care. Among these, 96 (13%) had a first trimester abortion, 12 (2%) moved away to other regions, 18 (2%) had multiple pregnancy, 87 (11%) presented after 12 weeks' gestation. 558 (72%) women met the study inclusion criteria, being under 12 weeks' gestation with singleton pregnancy.

Among these 558 women there were 108 (19%) women with autoimmune thyroid antibodies, 43 primiparous, and 65 multiparous. By taking away these 108 women, there remained 450 women without autoimmune thyroid antibodies defining the study group.

Regarding the distribution of maternal age and parity status (primiparous versus multiparous), the study group and control group were almost identical. In the study group, 39.8% were primiparous versus 39.2% in control group, and 60.2% were multiparous in study group versus 60.8% in control group. Dictated by body mass index (BMI), 61% of the primiparous group had normal weight at first consultation, and 39% were overweight or obese. Of the multiparous group, 60% had normal weight at first consultation and 40% were overweight or obese. There was a similar mean weight gain of 10–15 kg for primiparous and multiparous in pregnancy.

Peripartum outcome and fetal outcome resulted as follows: 68% of primiparous and 73% of multiparous had vaginal delivery within the study group. Cesarean section occurred in 34% of the primiparous (15% were elective, and 19% were emergency cesarean section) and in 28% of the multiparous (17% were elective, and 11% were emergency cesarean section). Pregnancy-induced hypertension occurred in 9 (2%) women of the study group (5 primiparous and 4 multiparous). Data from 2 women (1 primiparous and 1 multiparous) are missing. In the study group 22 (4.8%) women had breech presentation at term (11 were primiparous and 11 were multiparous). 

Data for Apgar score and arterial umbilical cord pH were almost complete. There is data missing for 2 Apgar scores and 8 arterial umbilical cord pHs. An Apgar score below 3, as a sign of impaired vitality was not registered at all and only 2 (0.5%) newborns had an arterial umbilical cord pH below 7.00, demonstrating asphyxia.

Apart from preterm births that were all referred to a pediatric hospital, further 35 newborns were referred to a pediatric hospital, 18 (11%) born by primiparous, and 17 (6%) born by multiparous.

**Figure 1:**
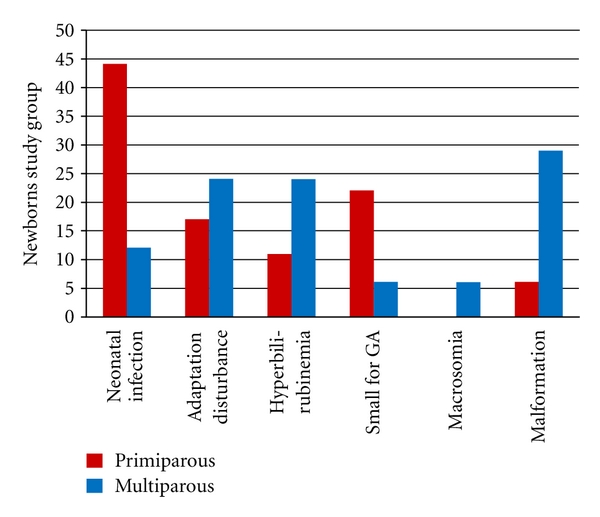
Percentage distribution of the reasons for referral of newborns in a paediatric hospital (*n* = 35). Preterm births are not shown.

Reasons for newborn referral to a pediatric hospital are listed in Figure 1.

**Figure 2:**
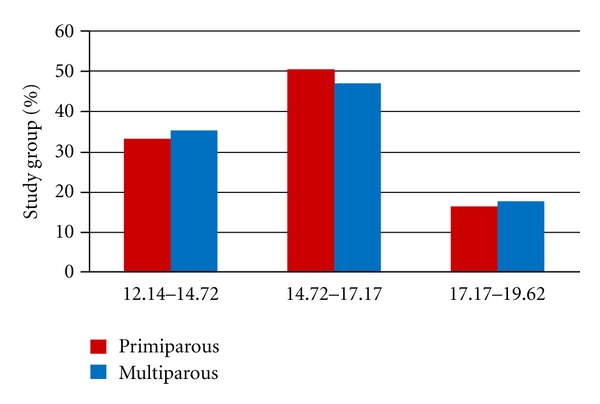
Percentage distribution of maternal fT4 serum level at first consultation subdivided into lower third, middle third and upper third of the reference range for nonpregnant women (*n* = 450).

Thyroid status of the study group is presented as shown in Figure 2. Only 16% of primiparous and 18% of multiparous had fT4 serum level in the high normal reference range prescribed for nonpregnant women at first consultation.

Preterm birth rate was first evaluated for the whole study group and separately depending on parity status, primiparous versus multiparous.

In the study group there were 20 preterm births (4.4%), 13 in primiparous (7.3%) and 7 in multiparous (2.6%). The gestational age of the preterm deliveries is shown in Table 1.

70% of preterm births from the study group were spontaneous preterm births caused by spontaneous preterm labor or preterm premature rupture of the membranes and 30% of preterm births were medically indicated by pregnancy-induced hypertension, HELLP syndrome, ovarian tumor, and acute pancreatitis concurring with obesity.

Preterm birth rate in the study group was 4.4% (99%-CI 1.9%–6.9%) versus 7.1% in the control group corresponding to a reduction of 38%. The subgroup analysis, according to parity status showed a preterm birth rate for primiparous in the study group of 7.3% versus 7.6% in the control group, and for multiparous a preterm birth rate of 2.6% in study group (99%-CI 0.1%–5.1%) versus 6.7% in the control group, respectively (Table 2). 

Thus, the reduction of preterm birth rate by maintaining maternal fT4 serum level in pregnancy in the high normal reference range prescribed for nonpregnant women is effective in multiparous but not in primiparous. The preterm birth rate dropped by approximately 61% in the multiparous of the study group.

Since there was no fixed dosage for L-T4 therapy and patients were advised to maintain the dosage unchanged in case of intolerance when dose augmentation had to occur, side effects were rarely noted. Nevertheless, if patients experienced adverse reactions, the L-T4 dose was reduced. Moreover, undesirable side effects for the fetus and newborn such as tachycardia or other signs of induced hyperthyroidism did not occur at all and were not recorded during cardiotocographic controls. Due to the variable L-T4 dose and the intake regime, L-T4 was well tolerated by pregnant women and complaints such as palpitations, tachycardia, and other undesirable clinical signs of hyperthyroidism were very rare and transient. However, pregnancy-associated nausea was more pronounced, predominantly in the first trimester.

## 6. Discussion

To our knowledge this is the first study presenting an essential benefit for reducing preterm birth rate in euthyroid multiparous women without thyroid autoimmune antibodies by keeping maternal fT4 serum level in the high normal reference range prescribed for nonpregnant women with L-T4 substitution. The preterm birth rate for multiparous in the study group declined by 61%. Primiparous did not benefit from L-T4 therapy. Possibly, the pathomechanism of preterm birth is essentially different among primiparous compared to multiparous, which would imply the necessity of different therapeutic strategies.

These results are in contrast to two other studies. Casey et al. found no mentionable risk for pregnancy outcome if the fT4 serum level was found to be in the lowest third of the gestational age specific reference range. Clearly, Goldman et al. recently compared pregnancy outcome in women with subclinical hypothyroidism and in women with hypothyroxinaemia defined as fT4 serum level below the 2.5th percentile. They failed to determine a link between subclinical hypothyroidism and adverse pregnancy outcome and hypothyroxinemia was not associated with the majority of pregnancy complications [21, 22].

In this study, a high maternal fT4 serum level decreased preterm birth rate in multiparous by yet unknown mechanisms. In some studies, it has been shown that human chorionic gonadotropin (hCG) plays a part in maintenance of uterine quiescence in the third trimester, and hence could be an endogenous tocolytic agent. HCG exerts a potent myometrial relaxant effect in human myometrium in the third trimester and inhibits preterm delivery in animals. Thyroid hormone stimulates the synthesis of hCG and the level of serum thyroid hormone is a positive regulator of serum thyrotropin bioactivity. Due to the fact that TSH and hCG share a certain similarity, being members of the same glucoprotein family, it appears possible that serum thyroid hormones can also positively regulate hCG bioactivity, which in turn increases the biological effects on the myometrium until parturition [1, 4, 14, 23].

The study group received L-T4 substitution to avoid a low maternal fT4 serum level for an optimal fetal brain development. The prevalence of low maternal fT4 levels is probably 150–200 times more common than congenital hypothyroidism [24, 25]. It was demonstrated that if maternal serum fT4 is low, fetal T3 levels in the brain will be low even in the presence of normal maternal and fetal serum fT3, suggesting that both T3 and T4 in the fetal brain are dependant on maternal fT4. Low maternal fT4 serum levels could have detrimental effects on fetal brain development. In animal experiments, it was demonstrated that even a modest and transient decrease in maternal fT4 resulted in altered brain histogenesis and cytoarchitecture of the fetal cerebral cortex [26, 27]. Various observational studies have shown that maternal subclinical hypothyroidism and low fT4 serum levels caused a significant decrease in IQ scores of the progeny. [8–11, 28–36]. In contrast to these findings, only one study failed to find an association between maternal thyroid function and cognitive test scores in children [37].

Handling thyroid function control by assessing only fT4 for a goal-oriented value as was done in this study and which is similar to controlling central hypothyroidism carries the risk to over dosage L-T4 and to provoke potentially metabolic hyperthyroidism. The usual assessment of an adequate L-T4 dose in replacement therapy is done by determining TSH and fT4 levels, preferably in a blood sample taken before ingestion of the subsequent L-T4 dose [38]. In nonpregnant states it takes 6–9 weeks and more to normalize a suppressed TSH value typically found when initiating L-T4 administration. As a consequence, it has been shown that patients taking 100 to 150 *μ*g/d of L-T4 have a nearly 50% probability that serum TSH will be undetectable [39]. Determining TSH below an interval of 6–9 weeks would be likely to result in a suppressed TSH level but would not necessarily be accompanied by an fT4 serum level in the upper third of the reference range [40].

Furthermore, L-T4 replacement therapy is commonly associated with supraphysiologically high fT4 and low fT3 serum levels, in general without hyperthyroid symptoms. Since fT3 serum level is maintained within normal limits by decreased peripheral conversion of the prohormone L-T4, supraphysiological fT4 serum levels are not considered to be harmful. Therefore, supraphysiological serum fT4 levels in patients taking L-T4 are not necessarily accompanied by clinical consequences, if there are no signs or symptoms of clinical toxicity. Clinical experience with pregnant women on TSH suppressive thyroid therapy after thyroid cancer treatment does not appear to indicate any complications [41, 42]. Silva and Larson have shown that T4 is preferentially converted to T3 in the pituitary gland to a greater degree than in other tissues, so that its TSH suppressive effect is greater than its metabolic effect. In other words, exogenously administered L-T4 suppresses both the pituitary gland and the thyroid gland as well [43].

In contrast to supraphysiological fT4 serum concentration seen in L-T4 replacement therapy, hyperthyroidism is defined as an excessive thyroid hormone production due to thyroid overactivity. The vast majority of cases of hyperthyroidism in pregnancy are induced by Graves' disease, toxic adenoma, or thyroid hormone resistance, where the negative feedback mechanism no longer functions. In these pathological conditions the overactive thyroid gland secretes both the metabolically inactive prohormone T4 and the metabolically highly active hormone T3 causing multiple symptoms. In Graves' disease, the intrathyroidal type-II deiodinase (D2), which activates thyroid hormone, has a 50 to 150 fold higher activity than in placenta and contributes significantly to the intrathyroidal T3 production and secretion [44]. Hyperthyroidism caused by Graves' disease and toxic multinodular goiter, high T3 concentrations are the result of excessive production and release from the thyroid gland and not of peripheral deiodination [45]. This explains why patients with Graves' disease or with toxic adenoma present symptoms of hyperthyroidism in contrast to patients taking L-T4 in a TSH suppressive dose. Additionally, in replacement therapy with a TSH suppressive L-T4 dose, T3 is derived completely from peripheral monodeiodination in the liver, kidney, or muscle because the thyroid gland is also suppressed. To achieve physiological levels of T3 in humans treated with L-T4, it is necessary to maintain fT4 levels at the higher end of the normal range [46, 47]. Only under pathological conditions, such as massive metastatic follicular thyroid cancer has T3 thyrotoxicosis by increased conversion of administered L-T4 been described [48]. L-T4 replacement therapy is associated with supraphysiologically high fT4 and low fT3 serum levels without hyperthyroid symptoms, since the fT3 serum level is maintained within normal limits by decreased peripheral conversion of the prohormone L-T4. This metabolic variance constitutes a fundamental difference between endogenously produced thyroid hormones T4 and especially T3 and exogenously administered thyroid hormone like L-T4. L-T4 has a safe latitude in dosage and even after massive acute ingestion only minimal symptoms are seen as the peak T3 level does not exceed the upper reference range limit, while fT4 and the metabolically inactive rT3 show very high serum levels [41, 43, 45, 47, 49–56].

Thyroid hormones are the most prescribed drugs worldwide and are considered to be safe. Relevant adverse effects are usually manifested in older patients. Disputable side effects are the risk of atrial fibrillation in patients with intrinsic heart disease and increased bone loss in postmenopausal women [57, 58].

Since there was no fixed dose regimen for L-T4 therapy in the study group, patients were advised to maintain their dosage unchanged in case of intolerance, and when dose augmentation proved necessary, side effects rarely occurred and in none L-T4 had to be discontinued. Nevertheless, if patients experienced adverse reactions the L-T4 dose was reduced. Moreover, undesirable side effects for the fetus and newborn such as tachycardia or other signs of induced hyperthyroidism did not occur at all and were not recorded during cardiotocographic controls. However, pregnancy-associated nausea was more pronounced, predominantly in the first trimester in only a few women. By reducing the L-T4 dosage, pregnancy-associated nausea resolved.

## 7. Conclusion

In summary, thyroid hormone replacement therapy aiming at holding maternal fT4 serum levels in the upper third of the reference range prescribed for nonpregnant women and controlling this therapy by determining fT4 seems to be safe and to have beneficial effects for both mother and fetus. In all probability it will reduce preterm birth rates in multiparous. These results have yet to be confirmed by further prospective randomized studies.

## Figures and Tables

**Table 1:** Numbers of preterm births plotted on gestational week and parity status the study group (*n* = 450).

Weeks' GA	<31 + 6	32 + 0 − 33 + 6	34 + 0 − 37 + 0
Primiparous	1	3	9
Multiparous	2	1	4

**Table 2:** Percentage of preterm birth rates plotted on parity status in the study group and control group.

	Total	Primiparous	Multiparous
Study group	4.4%	7.3%	2.6%
Control group	7.1%	7.6%	6.7%
